# Inclusive Communication Model Supporting the Employment Cycle of Individuals with Autism Spectrum Disorders

**DOI:** 10.3390/ijerph18094696

**Published:** 2021-04-28

**Authors:** Michał T. Tomczak, Joanna Maria Szulc, Małgorzata Szczerska

**Affiliations:** 1 Faculty of Management and Economics, Gdańsk University of Technology, 80-233 Gdańsk, Poland; 2 Huddersfield Business School, University of Huddersfield, Huddersfield HD1 3DH, UK; j.szulc@hud.ac.uk; 3 Faculty of Electronics, Telecommunications and Informatics, Gdańsk University of Technology, 80-233 Gdańsk, Poland; malszcze@pg.edu.pl

**Keywords:** autism spectrum disorders, neurodiversity, communication, recruitment, selection, onboarding, job retention, human resources management

## Abstract

Difficulties with interpersonal communication experienced by individuals with autism spectrum disorders (ASD) significantly contribute to their underrepresentation in the workforce as well as problems experienced while in employment. Consistently, it is vital to understand how communication within the employment cycle of this group can be improved. This study aims to identify and analyze the possibilities of modifying the communication processes around recruitment, selection, onboarding, and job retention to address the specific characteristics and needs of the representatives of this group. This qualitative study is based on 15 in-depth interviews conducted with 21 field experts, i.e.,: therapists, job trainers, and entrepreneurs employing people with ASD. The findings of this research informed the creation of an inclusive communication model supporting the employment cycle of individuals with ASD. The most important recommendations within the model that was created include the modification of job advertisements, use of less structured job interviews, providing opportunities for mentorship, and supportive and non-direct, electronically mediated communication. To apply the above-mentioned solutions and take full advantage of the talents of people with ASD, it is also necessary to provide tailored sensitivity and awareness training programs for their direct addressees as well as their neurotypical colleagues, including managerial staff.

## 1. Introduction

Employment is an integral part of life that provides individuals with a sense of financial security [1], determines self-esteem and social recognition [2], and has an impact on one’s wellbeing [3]. We now observe an expanding literature on the benefits of creating employment opportunities for people with disabilities [4,5,6], yet such individuals still experience a disproportionately high level of job insecurity, underemployment, and unemployment compared to workers without disabilities [7].

In this study, we focus specifically on people with autism spectrum disorders (ASD). ASD describes a set of developmental disorders including difficulties in interpersonal communication and social reciprocity with unusual repetitive behavior [8]. With the development of screening methods, the number of people diagnosed with these neurodevelopmental conditions is growing [9]. According to estimates, 1 in every 59 children in the United States may be characterized by this disorder [10]. Even though individuals with ASD often possess unique abilities such as extraordinary levels of analytical thinking [11,12], they are largely underrepresented in the workforce [13,14]. Statistics suggest that approximately 85% of people with autism are not in full time work [15] and 46% of adults with autism who are in employment are over-educated or exceed the level of skills needed for their roles [16]. Additionally, those who secure a job frequently experience isolation [17] and stigmatization [18] with negative knock-on effects on their well-being and mental health [19].

From the structural perspective on workplace stressors [20], there are many organizationally relevant factors that can cause discomfort in the context of work [21]. The Conversation of Resources (COR) theory [22] can help us understand the rationale behind such negative consequences being experienced by individuals with autism in the workplace context. Namely, the theory posits that a threat to our resources at work or failing to gain resources following resource investment will lead to higher levels of experienced stress. Because individuals with autism have different needs and require specific communication practices to contribute to organizational performance [23], it can be argued that the existing communication strategies generally used in the workplace context do not sufficiently address the specific needs of workers with ASD. For instance, difficulties associated with following social rules, understanding affect, reading facial expressions or the tone of voice, asking too many questions, or inability to ‘read between the lines’ (see: [24,25]) often mean that individuals with ASD experience problems with communication and social interaction with supervisors and coworkers [26,27]. Navigating such communication and interaction processes is often problematic, even before employment and begins, with the process of job searching and matching, through mastering the job application and interview process [24]. Such difficulties put the existing resources at risk and jeopardize the potential for gaining new resources. Ultimately, they can result in experienced stress and make individuals with ASD more vulnerable to the experience of loss spirals, i.e., further future losses [28]. In the light of largely atheoretical research on autism and employment (see: [29]), to the best of our knowledge, only two studies to date used COR as a theoretical framework to explore the experiences of employees with autism. Hayward et al. [30] used COR theory to demonstrate that employees with autism have only limited resources to cope with social-communication demands. In their subsequent research, the authors further used the theory to suggest that workplace relationships are the key resource to help employees with autism cope with such demands and manage organizational stress [31].

In this research, we therefore use the COR theory as a framework facilitating our understanding about the resources that employees with ASD need to cope with communication demands. Specifically, we pay attention to the resources needed to improve the communication processes around recruitment, selection, onboarding, and job retention, which can be perceived as a source of stress. These four stages are crucial elements of employment success for individuals in general, as well as those with ASD [11]. While there exists a significant body of literature investigating the effectiveness of HR practices across these four employment stages [32,33], it is not yet fully recognized that distinct categories of employees have different needs and require specific communication practices to contribute to organizational performance [23]. More specifically, because the communication deficits inherent in ASD may cause adults with autism to experience more stress [34], we need tailored communication strategies around recruitment, selection, onboarding, and job retention to create the most positive outcomes for the health and well-being of individuals with ASD in the workplace context.

Based on the above arguments, the aim of this research is to understand how communication within the employment cycle of individuals with autism can be improved. Grounded in the findings from qualitative interviews with experts in the field of autism employment, we provide a model of inclusive communication aimed at supporting recruitment, selection, onboarding, and job retention of individuals with ASD. In doing so we contribute to the existing literature in several ways.

First, most existing research on ASD and employment focuses on the challenges related to the recruitment and selection processes of people with autism [35,36]. By exploring potential improvements in the onboarding and retention practices, we contribute to the lacuna of research addressing these subsequent stages where individuals with ASD are reported to suffer most from stress and ill mental health [19,37]. In doing so, we contribute to recent calls for more theoretical and empirical research in this area [38,39,40]. Second, by demonstrating that the same HR practices could be differently perceived by neurotypical employees and their counterparts with ASD, we address the call by Cafferkey et al. [41] to move away from universal HR as a route to positive employee outcomes. Rather, we facilitate a more accurate reflection of organizational reality for disadvantaged members of society [42,43]. Finally, this study represents a departure from the conventional take on disability. While we see an expanding literature on workplace diversity in gender, ethnicity, age, or disability [44], little emphasis is paid to neurodevelopmental and cognitive disability [45,46]. Our study adds to the existing limited research by scrutinizing how practices can be developed to support ASD-friendly communication in the workplace context [47]. Practically, our study can assist HR practitioners in developing a comprehensive approach to communication throughout the employment cycle that is tailored to specific needs of ASD individuals to generate a positive impact on their health and well-being, with potential knock-on effects on improved organizational performance.

## 2. Methods

The study was part of the research project aimed at gaining new knowledge about individuals with ASD as a group that had not been a common object of research so far in the context of professional work. The study was set from a qualitative perspective and based on the in-depth interview method. During the interviews, respondents were asked how the HR processes such as recruitment, selection, onboarding, and job retention, can be reorganized to support the employment cycle of individuals with ASD. Although an interview agenda was used to maintain consistency, respondents were encouraged to engage in more complex discussions relating to the themes that they perceive to be most important. Ten interviews were individual, four had dyadic character [48], and one of the interviews was also conducted with three interviewees. All 15 interviews were conducted by the first author over a period of 10 months in 2020. Data collection was carried out with a group of 21 respondents, during face-to-face meetings, as well as online meetings using Microsoft Teams software. Twelve interviews were carried out, recorded, and transcribed in Polish, and three other were carried out in English.

The research sample was non-random and created based on the snowball method. Respondents were experts in the field, i.e., professionals with expert knowledge on the specific needs and competencies of people with ASD in the context of professional work, including therapists, job trainers/consultants, entrepreneurs employing people with ASD, and a parent of an adult jobseeker with ASD. The research sample consisted mainly of respondents from Poland, as well as from Canada, Australia, and Spain. The details of the research sample are presented in Table 1.

Each interview lasted approximately 35 min on average (shortest interview: 10 min; longest interview: 62 min). The transcription yielded a total of 103 pages that were analyzed using Template Analysis [49] and following a set of guidelines outlined by Brooks et al. [50]. The basic element of this technique is a coding template, which was developed based on a subset of data, which was subsequently applied to further data and revised and refined in the light of careful consideration of each transcript. Because the codes from the initial template were not rigid coding categories, but were rather provisional codes open to modification, when inadequacies in the initial template were discovered, modifications in the form of insertions, deletions, mergers, or changes to the scope of existing codes were made to allow for a comprehensive representation of data. Ultimately, the higher-order codes were defined to reflect each of the four stages of the employment cycle and lower-order codes pertained to the specific recommendations we discuss in the next sections. Once the initial template was developed to its final form, i.e., no new themes could be identified, it was applied to the full dataset and served as the basis for the writing up of findings. The first author was assigned primary responsibility for creating, updating, and revising the codes [51], and all authors engaged in intensive discussions to reach a consensus on the final coding template as an agreement goal.

## 3. Results

As a result of the analysis of empirical data obtained in the course of interviews, a set of recommendations was created to improve the way of communicating, bearing in mind the specific needs of neurodiverse people. The recommendations presented and discussed below include four HR areas: recruitment, selection, onboarding, and job retention (see Table 2).

### 3.1. Recruitment

The findings of this study suggest that instead of looking for the ‘perfect’ employee, the recruitment stage should be focused on looking for someone who can perform high-quality work with an appropriate level of support and in optimal working conditions. To facilitate the process of applying for jobs for candidates with ASD, recruiters should aim to create a neurodiversity-friendly job advertisement, where the content of the announcement is written in plain language and jargon and unnecessary requirements regarding qualifications are avoided. This is illustrated in the following representative quotation by one of the respondents:

*‘So definitely, first things first, it’s changing job advertisements, having them actually say what you usually do day-to-day. So no jargon, no unnecessary qualifications. Don’t state things like ‘you need to be a good person who can communicate or work in the team’ […]. If whether you can communicate or work in a team well is not an indicator of someone doing well on the job.’*

It was further found that job advertisements should contain clear information about the job position needs, the scope of duties and responsibilities, and a precise definition of daily tasks. The layout should be simple and composed of basic, not flashy colors. The content of the advertisement should be precise and understandable. Interestingly, it was reported that a typical job advertisement can be transformed into a request to solve a specific problem. In this way, the process of recruitment becomes concerned with choosing the best solutions instead of the best CV.

### 3.2. Selection

The findings from this study imply that the effective process of selection of individuals with ASD should employ competence-based assessment. Focusing on skills as opposed to a job fit was reported to ‘be less biased’. Additionally, it was commonly suggested that the emphasis during the selection process should be placed on current offerings of the candidates as opposed to their past behaviors. This perspective is illustrated by the quote from one of the respondents:

*‘Generally, we have a more patient recruitment process where we look at skill-based activities, not work experience. We don’t have interviews where you are required to sell yourself in 10 or 15 min. With us you have four weeks to build a relationship and to have us fully understand the individual. So, I think, work experience and work simulated tasks instead of selling yourself.’*

It was further reported that job interviews should be conducted in a friendly atmosphere where candidates can present all their possessed skills. It can be achieved through designing less structured interviews and using short and relevant verbal instructions. Interview questions should be detailed, clearly formulated, and precise, and abstraction and ambiguity should be avoided. Furthermore, the study participants commonly reported that it is necessary to provide a clear timeframe to conduct the task or respond to the questions during an interview. Practical skills tests, including gamification-based solutions, can also be effective in the process of selection within a group of individuals with ASD. If the organization does not have sufficient experience in recruiting neurodiverse talent, it can seek to facilitate the process with the assistance of a supported employment agency, or a specialist with an expert knowledge on what to expect and how to place accents during interviewing candidates with ASD.

### 3.3. Onboarding

Onboarding is another vital process that, as in the case of recruitment and selection, may be more difficult for individuals with ASD compared to their neurotypical counterparts. The following representative quote from one of the participants demonstrates that appropriate arrangement of the adaptation process by the employer is of key importance here:

*‘If the business or the employer was professional in their onboarding, they would say “how do I get the most productive outcomes from this new employee?”, and they would structure the way that that employee comes into employment.’*

The findings of this study suggest that the process of onboarding of employees with ASD should be preceded by ‘capacity building’ training programs for all employees, including management. Such programs should be aimed at promoting the acceptance of neurodiverse people as fit and valuable employees to ultimately support an inclusive environment and diversity climate. It is also worth providing the support of an experienced employee (buddy, mentor, job coach, etc.). Such a person would provide the necessary assistance with the introduction of new duties or clarifying doubts, especially in the difficult first days in a new job. New-starters with ASD should also be highly encouraged not to hesitate to ask for help if required, to talk about their needs, and to provide feedback on the adaptation process. Our findings further indicate that beyond clearly communicating the scope of duties for a new employee, the process of onboarding should also include an explanation of any unwritten rules of the workplace based on organizational culture, e.g., frequency of breaks, dress code, or organizational habits. To organize and structure the adaptation process, various types of onboarding checklists and written manuals or guides may also be used. Throughout the duration of the onboarding process, its participants should be regularly encouraged to share their observations and opinions about its course and to inform about their possible needs.

### 3.4. Job Retention

Effective recruitment, selection, and onboarding do not guarantee the employment success of neurodiverse individuals. The subsequent stage that should be addressed by organizations is the process of retaining such individuals in the workplace context. The findings reported in this research suggest that appropriate arrangement of communication methods within the organization may help with this process.

First, messages that are more understandable and legible can result in the communication process itself being less stressful for people with ASD. Consistently, our findings suggest that organizations should consider supporting their employees with ASD with non-direct and electronically mediated forms of intra-organizational communication, using e-mails, instant messaging, chatbots, online communicators, or online platforms. This will be a significant convenience, especially for people for whom intense verbal contact is a challenge. Moreover, this type of communication seems to be easier to measure, is more careful, and does not require an immediate verbal response. Another advantage of written communication is that it promotes the formation of more balanced and attentive messages.

Our findings further imply that meetings should be organized within small groups and take both stationary and remote forms using online platforms. The frequency of meetings should depend on individual preferences, but regularity and repeatability should be provided. As for the organization of work, a clear chain of command should be specified, such as contact with one person only (e.g., line manager). In the message content area, concise and precise messages should be used, as well as clear instructions, leaving no doubt as to what the task is, how to complete it, when it needs to be completed, and who is the person to approach with questions. It is also worth making sure if the information was clearly understood by the recipient. Among the valuable solutions presented by the study participants, there are also: introducing structure where possible, providing a task sequence, and sharing information about changes, in advance if possible. Moreover, verbal instructions should be followed by the written form. Visual forms such as visual job schedules or instructional pictures were also reported to be effective. It can also be useful to provide a written agenda before the meeting and minutes after the meeting. Feedback should be direct but sensitive, possibly avoiding the unnecessary emotions influencing or disturbing the communication process.

Importantly, the above activities must be fully supported by neurodiversity-aware managers, as one respondent stated:

*‘I think support and understanding from the employer is most important. There would be no job retention if the employer or the management in the team was not supportive and understanding. They need to provide a caring environment for autistic employees to make sure they feel comfortable.’*

## 4. Discussion

The aim of this study was to identify and analyze the possibilities of modifying communication processes in the area of recruitment, selection, onboarding, and job retention in terms of the needs of employees with ASD. We built upon previous research on supporting people with ASD in employment [52,53,54,55,56]. The focus of this study, however, is on changes that employers can make to their HR practices, rather than the onus being entirely on the individual with autism to adapt to the existing practices. Based on the interviews with experts in ASD employment, we developed an inclusive communication model supporting the employment cycle of individuals with ASD and potentially minimizing the experienced stress levels by this group of employees. Because individuals with ASD are stress-vulnerable and experience high self-perceived stress levels [57] due to the problems they face with coping with stressful situations [58,59], the oppressive effect of stress is highly amplified for this group [47]. Consistently, finding ways to improve the communication process at each stage of the employment cycle to help to minimize perceived stress levels through regeneration of resources [22] appeared to be particularly vital.

Our model is based on a list of potential improvements in the communication process at each individual stage of the employee cycle. More specifically, we demonstrated that the employment cycle of individuals with ASD should start with reorganizing recruitment and selection processes through the modifications of job advertisements and the use of less structured job interviews. Such practices appear more effective and less stressful to people with ASD, who often find it problematic to boast about their professional experience. The subsequent onboarding stage would benefit from the support of a mentor, buddy, or job trainer who can boost the confidence of employees with ASD and provide reassurance in stressful situations. To support the job retention of individuals with ASD, organizations should further consider organizing day-to-day work with the use of precise messages, direct but sensitive feedback, and limiting emotions during the communication process, as well as using written and non-direct, electronically mediated communication forms to support a stress-free process of communication. Importantly, the improvements and activities presented above should be preceded by tailored sensitivity and awareness training programs for individuals with ASD as well as their neurotypical colleagues, including managerial staff.

The obtained results are consistent with the findings of previous studies in the area of workplace accommodations and technology-aided interventions [29,60], matching workplace success strategies for employees with ASD [39], perspectives on well-being of neurodiverse employees [43], and employment success factors [61], including recruitment and selection [32]. The results confirm the need for promoting supported and customized employment [62] and prove the importance of modifications and support in the area of the communication process improvement as one of key elements within the vocational assistance of individuals with ASD [30,38].

Our model of inclusive communication for prospective and current ASD employees not only highlights potential adjustments that could be employed by HR departments to achieve optimal employment outcomes, but it also enables a better theoretical understanding of the conditions under which high performance of individuals with ASD could be achieved. We partly answer the calls for existing mainstream HR research to stop treating employees as an undifferentiated mass [63], and we acknowledge the importance of differentiated HR practices that capture the unique needs of diverse groups of employees [41]. This appears to be particularly important against a backdrop of calls for more theoretical and empirical research exploring the inclusion of individuals with autism into employment [38,43], and the majority of research efforts not focusing beyond the first stages of the employment cycle (e.g., [36]).

An important next step is for organizations to employ a change in perspective where the onus of employment interventions is not predominantly targeted at people with autism but on the changes that employers can make to enable them to thrive in the organizational context [57]. Indeed, the reported research contributed to meeting the urgent need to explore ways in which the successful employment of individuals with autism can be facilitated. This, in turn, should become the starting point for further research, where the introduced solutions would be tested in real work environment conditions.

Despite its strengths, the reported study is not free from limitations. First, the current findings are based upon the perceptions of people labelled as experts in autism employment, and only one of these experts was diagnosed with ASD. However, this precludes a more holistic understanding, including the perspectives of individuals with ASD and their lived experiences. Consistently, there is a need for future research to better understand the factors influencing successful employment for autistic adults in the labor market from their own perspective. Second, our sample consisted only of five employers. This is due to the limited access to the representatives of companies successfully employing people with ASD. In Poland, where most of the interviews were conducted, there are only a few employers of this type, as neurodiverse employment is only starting to gain popularity. We therefore conducted some further interviews with therapists as field experts who specialized in working with adults with ASD. Third, we are aware that people with ASD are not a homogeneous group. In our study, we focused mainly on the possibilities of developing solutions addressed to high-functioning individuals with ASD, including people with Asperger’s Syndrome, i.e., those who are independent and able to apply for a job on their own, and then maintain it successfully. They often have academic qualifications and professional competencies at a high level, but due to the difficulties associated with specific cognitive styles, they face problems with finding and maintaining employment, which is more stressful for this group compared to the general population [64]. Future research efforts should be focused on the wider population of individuals with autism, including those who are considered to be low-functioning. Fourth, while the preliminary nature of the reported study allowed us to gain insights into effective communication processes, future research would now benefit from testing the proposed solutions, for instance, during field experiments. Similarly, there is an urgent need for future research to evaluate the effectiveness of such accommodations. Further research in this area would enable us to see what particular adjustments work for different individuals and roles and what impact this may have on organizational performance [49]. In all these endeavors, interdisciplinary collaborations can lead to developing integrated and comprehensive solutions to the persistent problems faced by an autistic minority.

## 5. Conclusions

In an increasingly challenging environment for employability and organizational sustainability, a more nuanced understanding is needed of how employers and Human Resource departments can support individuals with ASD and facilitate opportunities for effective communication at all levels of the employment cycle while promoting a more customized well-being agenda. Given that individuals with ASD experience challenges with communication, they may possess fewer resources to cope with complex communication processes in the workplace, which ultimately results in higher levels of perceived stress. Meanwhile, without doubt, more holistic and sustained interventions targeting inclusive organizational cultures with a diversity climate in general are vital to provide appropriate support for individuals with ASD; in this article we provided recommendations specific to improving the communication process at all stages of the employment cycle. We believe this to be a first step towards developing integrated and comprehensive solutions to the persistent problems faced by autistic minorities that will also be to the benefit of all workplace parties.

## Figures and Tables

**Table 1:** Detailed Information on the Research Sample.

Characteristics		Number of Respondents
Gender	Female	14
	Male	7
Country of origin	Poland	17
	Canada	2
	Spain	1
	Australia	1
Role	Therapist	10
	Job trainer/consultant	5
	Employer	5
	Other	1

Source: Own study.

**Table 2:** Possible Solutions Based on Improved Communication Mode Supporting Recruitment, Selection, Onboarding, and Job Retention of Employees with ASD.

Support Area	Possible Communication-Based Improvements
Recruitment	Job advertisements written in plain language, avoiding jargon and unnecessary requirements regarding qualifications, with clear information about the requirements, scope of duties and responsibilities, a precise definition of daily tasks
	Simple layout, using basic colors
	A request to solve a given problem instead of a typical job advertisement, and choosing the best solutions instead of the best CV
Selection	Less structured job interviews
	Verbal instructions, short and to the point
	Detailed questions, properly formulated, precise, without abstraction, without ambiguity
	Providing clear time frames to conduct the task
	Practical skills tests, including gamification-based solutions
	Recruitment with the support of a specialist or an expertly trained person (who has expertise knowledge on what to expect and how to place accents)
Onboarding	Promoting the acceptance of neurodiverse people as fit and valuable employees, supporting an inclusive environment and diversity climate
	Providing support of buddy, mentor, job coach
	Encouraging asking for help if required
	Encouraging feedback and informing about own needs
	Using onboarding checklists, manuals, and guides
	Explaining any unwritten rules of the workplace (breaks, dress code, etc.)
Job retention	Non-direct, electronically mediated communication (e-mails, instant messaging, chatbots, online platforms, etc.)
	Meetings organized in small groups (both remote and stationary)
	The frequency of meetings depending on individual needs but with regularity and repeatability
	A clear chain of command, contact with one person, the line manager
	Using concise and precise messages, clear instructions (what the task is, how to complete it, when it needs to be completed by, who to approach if there are questions)
	Introducing structure where possible, providing task sequence
	Checking if the information was clearly understood
	Providing information about the change in advance
	Verbal instructions followed by written form
	Written communication using visual forms (visual job schedules, instructional pictures)
	Written agenda before the meeting and minutes after the meeting
	Providing direct but sensitive feedback
	Avoiding emotions that influence the communication process

Source: Own study.

## Data Availability

The dataset used and analyzed during the current study is available from the corresponding author upon request.
